# The Washington Needle Depot: fitting healthcare to injection drug users rather than injection drug users to healthcare: moving from a syringe exchange to syringe distribution model

**DOI:** 10.1186/1477-7517-7-1

**Published:** 2010-01-04

**Authors:** Dan Small, Andrea Glickman, Galen Rigter, Thia Walter

**Affiliations:** 1 PHS Community Services Society, 20 West Hastings Street, Vancouver, BC, V6B 1G6, Canada; 2 Department of Anthropology, University of British Columbia, 6303 NW Marine Drive, Vancouver, BC, V6T 1Z1, Canada; 3 Union of BC Indian Chiefs, 500 - 342 Water Street, Vancouver, BC, V6B 1B6, Canada; 4 PHS Community Services Society, 20 West Hastings Street, Vancouver, BC, V6B 1G6, Canada; 5 Life is not Enough Society, 42 Blood Alley Square, Vancouver BC, V6B 1C8, Canada

## Abstract

Needle exchange programs chase political as well as epidemiological dragons, carrying within them both implicit moral and political goals. In the exchange model of syringe distribution, injection drug users (IDUs) must provide used needles in order to receive new needles. Distribution and retrieval are co-existent in the exchange model. Likewise, limitations on how many needles can be received at a time compel addicts to have multiple points of contact with professionals where the virtues of treatment and detox are impressed upon them. The centre of gravity for syringe distribution programs needs to shift from needle exchange to needle distribution, which provides unlimited access to syringes. This paper provides a case study of the Washington Needle Depot, a program operating under the syringe distribution model, showing that the distribution and retrieval of syringes can be separated with effective results. Further, the experience of IDUs is utilized, through paid employment, to provide a vulnerable population of people with clean syringes to prevent HIV and HCV.

## Historical context of needle exchange

So, so you think you can tell heaven from hell,

Blue skies from pain.

Can you tell a green field from a cold steel rail?

A smile from a veil?

Do you think you can tell?

(Roger Waters; David Gilmour)

Needle distribution programs take place against the backdrop of public health. Public health has been a core part of medicine in Canada since before the establishment of the Canada Medical Act in 1912, and can be defined as a preventative approach to improving and maintaining the health of a population. The Canadian medical profession has a long history of protecting innovations in public health. The first president of the Medical Council of Canada, Dr. Thomas Roddick, initiated a campaign to establish a Canadian public health bureau as early as 1899 [1]. In the first national licensing exam of 7-10 October 1913, Public Health, or Hygiene and State Medicine as it was called then, was a key subject area on which the earliest physicians had to demonstrate competence in order to obtain their licensure for practicing medicine in Canada [2]. By 1929, the subject of this portion of the national qualifications exam was changed to Public Health and Preventive Medicine. Today, public health is still a subject on which all those individuals seeking medical licensure in Canada are tested. This paper describes the innovations of a peer-professional needle distribution program, where people with addictions deliver healthcare, under the umbrella of public health.

Needle distribution as a response to addiction related infections first came about in response to hepatitis B and C [3]. One of the earliest recorded needle distribution programs was launched by a pharmacist in Edinburgh in 1982 in response to an outbreak of hepatitis C [4]. At about the same time, a peer based organization of people living with addictions called upon the health authority in Amsterdam to initiate needle distribution to help curb the spread of hepatitis B [5]. Needle exchange in Canada also began in partnership with people who had direct experience in addictions.

Canada's first needle distribution program began in February 1989 as a health initiative to control the spread of HIV/AIDS [6,7]. The program was initially a 10-month pilot funded by the City of Vancouver during the period in office of Mayor Gordon Campbell who continued on in public service to become the Premier of the Province of British Columbia. A non-profit organization headed by former addict John Turvey, the Downtown Eastside Youth Activities Society (DEYAS), delivered the service along with a local health clinic, North Health Unit, who provided expert input from clinicians as required [6]. The program began with two staff.

At the beginning of the program, injecting drug users (IDUs) were limited to two syringes in attempts to prevent people from selling the sought after needles in order to purchase drugs. In the early stages of the program, exchange, that is, the provision of a used needle in order to obtain a clean needle, was encouraged but not compulsory [6]. During the first year of the program, the price of syringes for purchase on the street dropped from five dollars to one dollar per needle.

The injection of cocaine became a major obstacle to needle exchange with daily syringe limits and exacerbated the HIV epidemic in IDUs living in Vancouver. With the arrival of injecting cocaine in the 1990s, enforced exchanges and low limits on the number of syringes available in a given day, were a recipe for epidemiological disaster [8]. The relatively short duration of cocaine's effects meant an increased quantity of injections (i.e. an increased need for syringes) per day for users. At this time, the PHS Community Services Society (PHS), a non-profit organization based in Vancouver's Downtown Eastside (DTES), was the only organization in Vancouver, to provide unlimited amounts of needles to IDUs based on need as determined by the addict and not the agency. The PHS pursued this model of syringe distribution in spite of opposition from DEYAS at the time,

In the 1990s, DEYAS had a policy of limiting the number of syringes that IDUs could obtain in a single day and over the course of a week. Specifically, IDUs could obtain a maximum of 14 syringes per day, three days per week for a total of 42 needles per week [9]. If an individual was known to be living with HIV or HCV, then they were allowed to double this rate of exchange for a total of 84 needles per week. In addition, clients of the needle exchange were allowed to trade an additional five needles per day at each stop of the mobile needle exchange van. Bulk exchanges (more than one needle at a time) were only allowed at the fixed needle exchange and were not allowed at the mobile exchange vans. As well, there was a policy of "trading" meaning that addicts had to provide a used needle in exchange, or trade, for each clean needle that they provided through the "exchange". The needle exchange would allow for a single "loaner" syringe per person in case an IDU did not have a needle to trade.

Enforcing a trading system with a one-for-one exchange policy and limiting the amount of syringes obtainable was meant to obtain three objectives [9]. Firstly, the exchange system was meant to maximize the point of contact between the needle exchange staff and individuals with active addictions in order to develop rapport and facilitate opportunities for providing healthcare information as well as referral to treatment, detox, and counselling. Secondly, the exchange approach was meant to recover as many used needles as possible. Thirdly, an exchange approach with fixed limits was supposed to maximize the amount of clean needles in circulation while minimizing the amount of dirty needles available for re-use.

## The end of an era in needle exchange

In its final years of operation, the DEYAS needle exchange program experienced significant challenges. When DEYAS closed their long-term fixed site needle exchange but did not have a suitable replacement site, the PHS immediately provided a new needle exchange site and assisted in the acquisition of a municipal permit despite opposition from a local government that was hostile towards needle exchange.

After two decades of operation, the DEYAS needle exchange program ceased to operate in July of 2009. As a result, the PHS stepped up its efforts to stretch its existing resources to subsume the roles previously undertaken by DEYAS including the retrieval of used needles and increased mobile syringe delivery to IDUs throughout the city of Vancouver. Subsequently, the local health authority commissioned a review of needle exchange services and put all syringe distribution programs delivered by non-profit agencies, including those operated by the PHS, out to tender.

In an attempt to save the most important part of the services provided by DEYAS (i.e. primarily mobile syringe delivery, outreach, collection of discarded syringes, emptying needle boxes that have been deployed in the community), the PHS moved to fill this gap utilizing the existing infrastructure and capacity of the WND. The program had the capacity to provide the service immediately so that there was no service interruption. The organization did not have to purchase or rent a van; they already had one that was purchased by the health authority. They made use of a fully functioning location already funded for this very purpose. The existing coordinator at the PHS needle distribution program assumed the responsibilities of supervising the services formerly provided by DEYAS. Today, the program operates 24 hours with a fixed site as well as a mobile syringe delivery, retrieval and outreach service. The remainder of this paper focuses on the importance and urgency of keeping needle distributions (as opposed to exchanges) in operation as a public health measure.

## Out of the healthcare hurricane: context of the Washington Needle Depot

The PHS has been a provider and supporter of syringe distribution for 17 years. The PHS was the first housing agency in Vancouver to operate an "in house", fixed, needle distribution program in 1993, and the first HIV organization to receive funding for syringe distribution in BC. The Washington Needle Depot (WNP) opened as an extension of the organization's existing needle distribution services. The PHS was also the first organization to provide unlimited syringe distribution without the necessity of exchange. This was especially important during the HIV epidemic that exploded in the IDU population in Vancouver during the mid-1990's.

The organization has been a vocal advocate of the decentralization of syringe distribution and the distribution of clean needles through all community health centres in the region. The PHS has always argued for a fixed site for syringe distribution, open 24 hours per day, coupled with outreach needle distribution and retrieval in the DTES.

The WND operates in the Downtown Eastside (DTES) community of Vancouver, which is a densely populated and diverse urban neighbourhood. There is a high rate of poverty and a concentration of people with active addictions. There is a high rate of homelessness and inadequate housing. Thousands of low-income residents in Vancouver live in single room accommodation (SRA) hotels: tiny rooms (e.g. 140 square feet) where they share a bathroom and kitchen with dozens of other tenants. The ethnically mixed population includes a disproportionately high number of Aboriginal residents. Approximately thirty percent of the residents of the DTES are indigenous, 10 times the national average [10]. Recent studies demonstrate that youth and adult aboriginal drug users in the DTES have an elevated risk of HIV infection [11,12].

The WND emerged in its present location as part of a response to a healthcare and political crisis in Vancouver. On 31 May 2002, the Vancouver Police Department (VPD) shut down a satellite needle distribution program located on the corner of Main Street and Hastings Street in the DTES. This program operated under a tent, equipped with a humble table and two chairs purchased from a local department store. People with addictions, peer to peer volunteers, from the Vancouver Area Network of Drug Users (VANDU) and staff from the PHS sat each night to hand out harm reduction supplies (syringes, Band-Aids, condoms) [13,14]. Despite the fact that the health authority made needles available at several locations at that time, the needle exchange table was the only location providing service after traditional business hours.

The immediate result from the police closure of the needle distribution program was a significant reduction in the amount of needles distributed. Similar experiences occurred when the only needle exchange was shut in Victoria (the capital city of British Columbia) [14,15]. The shutting of the Victoria needle exchange resulted in a 23% reduction in syringes distributed. Reductions in the amount of syringes distributed due to closure of health programs leads to higher risk of deadly infections (e.g. HCV, HIV) in IDUs. In response to the closure of the Vancouver needle distribution program in 2002, the Centre for Excellence in HIV/AIDS, a department of St. Paul's Hospital and the University of British Columbia, submitted a letter to the Vancouver Police Board requesting that the police allow the exchange to be re-opened immediately to prevent an increase in risk for HIV and HCV infections due to the closure.

On 19 July 2002, the City of Vancouver and the Vancouver Police convened a meeting with the funder for the program, Vancouver Coastal Health (VCH), and the agencies delivering the service (PHS and VANDU). Further to the sudden closure and confiscation of the table, tent and needle exchange equipment, the police and city representatives argued that the actual needle exchange *table *did not have a municipal permit to operate. The police went on to state that they would not allow the peer-to-peer needle distribution program to commence until the VCH committed to re-designing the services available to IDUs at the street corner in question. Further, they demanded a written plan describing the longer-term vision for needle exchange for the City and a direct connection between needle exchange, treatment and detox. By having seized and closed the syringe distribution program itself, literally, enforcement officials were ironically attempting to dictate a specific agenda, arguably outside of their expertise, with regard to healthcare services in the neighborhood.

The meeting had a number of outcomes. The VCH made it clear that their organization did not want to break the law in any way and agreed to cease operating a fixed needle exchange at the corner until such a time that the permit was obtained. The City expressed concerns about the lack of a permit for the table. In the spirit of working with the police, both VANDU and VCH agreed to halt the program in its current configuration until the demands of the police were met. Needle exchange would continue with roaming peer-to-peer workers distributing needles from "fanny packs".

In contrast, the PHS was in marked dissention. The city permit process lays open healthcare programs like needle distribution for public debate in forums as part of the municipal process. In these cumbersome public forums, healthcare is politicized as opponents to needle exchange are given an opportunity use the municipal process to voice their opposition to the syringe distribution in general. In light of the research evidence presented by the Centre of Excellence in their communications on the matter, it appeared clear that roaming needle distribution was not as effective as a fixed exchange coupled with a roaming approach. In fact, there was some speculation that there would be a statistical likelihood of risk for one preventable HIV infection per night while the fixed site was closed at the corner. As a result of these factors, the PHS gave the VPD a deadline of 4:00 pm to return the table and allow the program to re-commence, or the organization would erect a new needle distribution table at the corner. Subsequently, several activists were lined up, including a number of public figures, who agreed to volunteer at the table and risk possible arrest. At the time, the PHS was forced to seek legal advice regarding possible charges such as being arrested for conspiring to save lives.

There was, as a result, some tension between the supporters of the program: the VCH, VANDU and the PHS. The hard-line approach of the PHS was in direct contravention of the wishes of VCH and VANDU both of which formally registered their protest to the PHS. Concurrently, the PHS made an immediate application for the described permit to the City. The front line city officials in the permits and licensing department examined the application with hilarity and contradicted the senior City management by stating that no such permit was required or even available. Further, photographs of tables without permits, crowding the sidewalks of Chinatown one block away, were presented to the City as part of an argument that no such permit was necessary. It increasingly appeared that the demand for a municipal permit was a charade to mask opposition to syringe distribution.

In the end, the VPD missed the deadline. The PHS dispatched a new table. Shortly after the PHS dispatched the tent and new custom-built table on wheels, without the unobtainable municipal permit, the VPD opposition collapsed. Subsequently, the PHS negotiated a contract from the VCH to provide a fixed site along with outreach patrols distributing and retrieving syringes. The program also while provided healthcare information and referrals to treatment and detox. Condoms were also distributed that were accessed by a broad population including survival sex workers. The PHS provided a free site for the program in the Washington Hotel as part of the organization's ongoing syringe distribution and retrieval services. The WND was born.

## Early indicators of the need to move from exchange to distribution

Critical examinations of needle exchange suggest that these programs need to be decentralized and flexible [16,17]. Early research in Vancouver, Canada suggested that needle exchange needed to be a part of a comprehensive program to address and reduce HIV and HCV incidence [17]. Vital to this comprehensive approach was a need to switch to a distribution model rather than exchange. Likewise, decentralization of syringe distribution was critical; needles needed to be available at many locations.

Many exchange programs have a rehabilitative focus: limiting the amount of syringes obtainable at one time in order to force multiple points of contact with people with addictions and to compel participants to become reliant on the programs. Needle exchange, in many cases, is seen as a doorway to referrals and counseling [8].Despite widespread cocaine use in Vancouver in the 1990s that necessitated considerable access to syringes (cocaine users have been known to require more than one dozen needles in a single day), needles were often limited and exchange policies were employed so that addicts had to provide a dirty needle in order to obtain a clean one. In some circumstances addicts would, presumably, be turned away because they had either reached their limit for the day or did not have a dirty needle to trade for a clean one. Early studies highlighted the limitations of needle *exchange: *embedding rehabilitative goals in that limit the number of syringes obtainable by an individual IDU.

Difficulty in obtaining syringes is a key risk factor for syringe sharing [8,18]. IDUs who obtain all the needles that they require are measurably less likely to engage in high-risk injection practices[18]. In fact, a significant portion of individuals who initiate use of syringe distribution programs report stopping syringe sharing altogether [19].What is required for maximum effectiveness are more, not less, needles. The difference between needle exchange and needle distribution is significant, two distinctly different healthcare initiatives, a topic that is addressed in the remainder of the paper.

## Effectiveness of needle distribution

HIV and HCV can be transmitted via infected blood traveling from one person to another through a shared needle. The basic approach to needle distribution is to provide IDUs with clean needles so that a new needle is used every time to avoid transmission of infectious diseases. As part of the program, drug users are educated about dangerous injection practices: (e.g. sharing needles). There is persuasive scientific evidence that needle syringe programs reduce the risk of HIV and HCV considerably. Further, credible data of any harmful consequences of these healthcare programs do not exist [3,19]. Syringe distribution is supported by a myriad of mainstream medical, scientific and government bodies including United Nations, the World Health Organization, United Nations Office on Drugs and Crime[20], the American Academy of Family Physicians[21], the American Medical Association[22], the U.S. Centers for Disease Control (CDC)[23], the U.S. National Academy of Sciences Institute of Medicine[24], American Society of Addiction Medicine[25] and the U.S. National Institutes of Health [26]. There is widespread consensus in the medical and scientific community regarding the effectiveness of distributing clean syringe equipment as made evident by an open letter written to the Office of National Drug Control Policy by Ranking Member Henry A. Waxman on behalf of the Congress of the United States House of Representatives Committee on Government Reform on 25 May 2005 (see additional file 1).

In response to the AIDS pandemic, the United Nations General Assembly unanimously adopted an imperative Resolution to address AIDS on 2 June 2006. In this resolution, the United Nations General Assembly unanimously and publicly declared the importance of harm reduction and needle distribution by reiterating that:

"...prevention of HIV infection must be the mainstay of national, regional and international responses to the pandemic, and therefore [we] commit ourselves to intensifying efforts to ensure that a wide range of prevention programmes that take account of local circumstances, ethics and cultural values is available in all countries, particularly the most affected countries, including information, education and communication, in languages most understood by communities and respectful of cultures, aimed at reducing risk-taking behaviours and encouraging responsible sexual behaviour, including abstinence and fidelity; expanded access to essential commodities, including male and female condoms and sterile injecting equipment; harm-reduction efforts related to drug use; expanded access to voluntary and confidential counselling and testing; safe blood supplies; and early and effective treatment of sexually transmitted infections;"[27] (p. 4).

## Psychosocial Engagement

**Figure 1:**
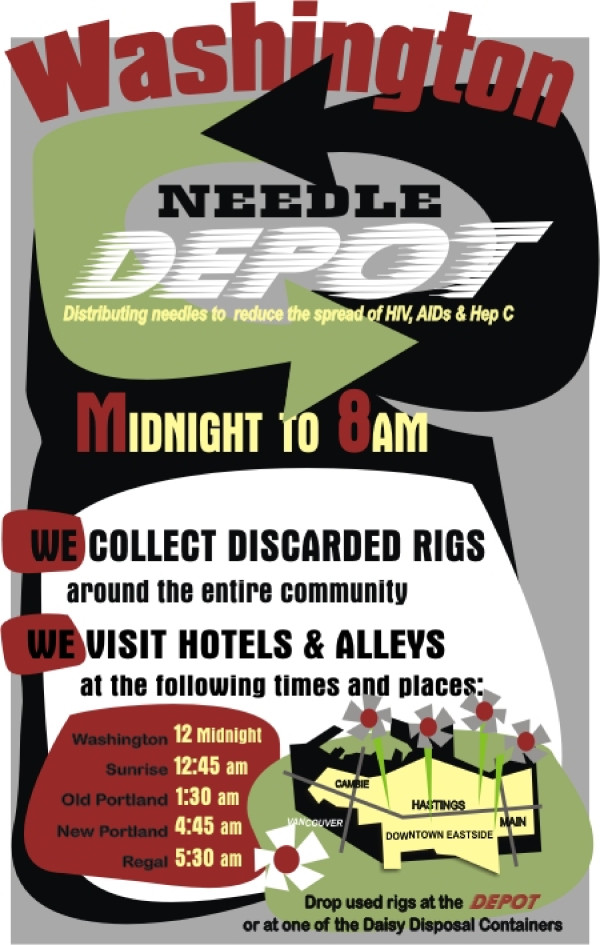
**WND educational poster**. A poster placed in the allies in Vancouver describing the services of the WND.

There is a difference between the cost of a needle that is delivered in the alleyway at 3:00 am and a needle that is available at a health clinic during business hours. Needles services that are delivered from 9:00 am to 5:00 pm as an adjunct to a given program are relatively easy to deliver as they are simply added onto to existing facilities. However, syringe distribution and retrieval that occur between 5:30 pm to 9:00 am are more challenging. These services require staff to be available at more challenging hours and in more challenging areas (e.g. the alleys and SRA hotels). It is precisely in these more difficult times and places that the WND operates and flourishes at a much lower cost than could be provided through a higher threshold, professionally based, healthcare institution. (See Figure 1)

Impediments to acquiring syringes are the prevailing risk factor for dangerous injection practices that can lead to infectious diseases HIV and HCV [18,28]. Needle distribution can have a dramatic impact: IDUs who receive all their syringes from a NEP are considerably less likely to share syringes [18,19]. By engaging street level IDUs in service provision through syringe distribution and retrieval, the WND represented a fundamental shift in the centre of healthcare gravity. Rather than simply receiving services, vulnerable IDUs could be actively involved in delivering them. This went one step further than being consulted about how to best deliver services to drug addicts to actually paying IDUs to deliver service. Additionally, this meant recognizing that their experiences provided them with a unique insight and ability to deliver peer-based harm reduction services, including being easy to approach for IDUs seeking services. IDUs often report seeking services at the WND because of familiarity and comfort with the peer workers.

People who still inject drugs can be involved in the program. In a "work-first" approach, traditional rehabilitation models are turned upside down: rather than forcing people to be "in recovery" before obtaining work; this program gives people work immediately as part of their recovery. In an "employment first" approach, work is a part of the initial recovery process. Rather than being the end destination in their recovery, involvement in salubrious activities like harm reduction services becomes one of the first steps in the road.

**Figure 2:**
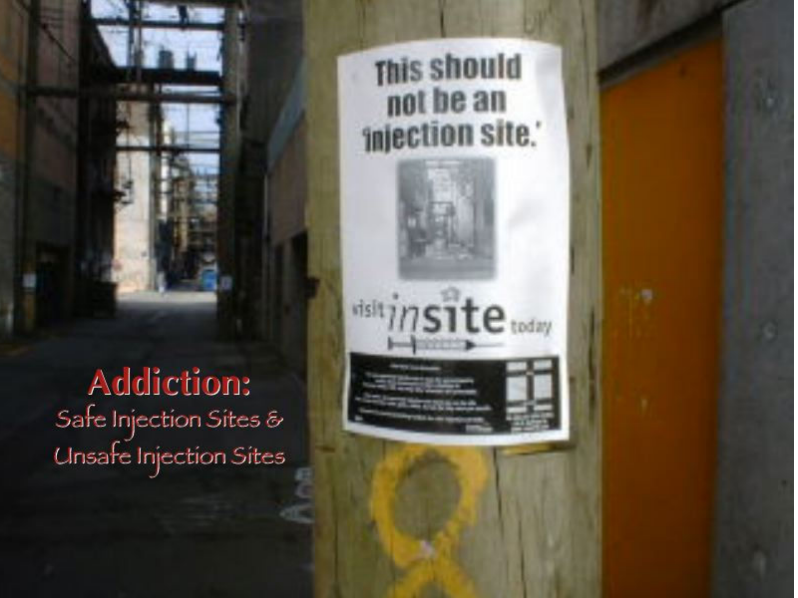
**Educational poster in an alley**. A poster placed by the WND in the allies in Vancouver describing a safer place to inject drugs under the supervision of medical personnel.

The WND provides a 'safe place' where people who have been barred from other service locations regularly attend. Discussions on politics, jail, and childhood happen regularly, along with conversation around harm reduction. People come and go all night, and sometimes disappear altogether, often seeking recovery, before returning again to the WND as a point of connection with the community. The outreach component of the program also allows for public education on a variety of other public health issues. The WND outreach workers, by way of example, place educational materials about treatment, detox, healthcare programs, referrals and harm reduction in alleyways frequented by IDUs (see Figure 2) The use of posters is an effective way to reach people who live below the poverty line who do not read newspapers or watch television.

The WND provides a range of low, medium and high threshold employment opportunities that range from pre-vocational skills training stipend positions all the way to full time employment in delivering harm reduction services. As of July 2009, there was a total worker pool of approximately 70 members, with varying levels of involvement. Some are solely dependent on the WND as their only source of income, and for some it is purely about giving back to their community. For many peer workers it is a four-hour relief from their daily struggle for survival, a place to socialize with peers and take a break from the street. The WND is also one of the only places to obtain work even for those who are physically or educationally challenged. Several workers are amputees, some have serious weight and heart problems, and some cannot read or write; all such challenges are approached with respect and a willingness to adapt and be creative. The PHS Program Coordinator oversees the service delivery, maintains delivery and retrieval statistics. They focus on removing barriers to service for marginalized IDUs while supporting and engaging a range of street level IDUs as participants in the program.

People with active addictions are recruited from the street level to engage in low threshold positions in syringe distribution and are signed up on a daily or weekly basis. Many people decide to volunteer after using the services themselves. A person can commit to one shift on a particular given day and be paid the same day. Jobs are distributed at bi-monthly meetings at the WND. Names are chosen by a lottery draw and work amounts on average to three or four, four-hour shifts per individual in a two-week period. These shifts currently operate between 8 am-12 pm and 10 pm-2 am, 7 days a week and are paid out in a cash stipend. These shifts are flexible as to when they should be deployed.

Higher threshold opportunities, though still within the low threshold continuum, are available for those individuals who have undergone a probationary period in the low threshold category. The Peer Supervisor position is available to a peer recognized for his or her hard work. Promoted to this position, the peer takes on more responsibility following which coordinators regularly observe noticeable improvements in the self-esteem of workers. The Peer Supervisors earn a liveable wage and receive a regular cheque. This has resulted in several peers who have been able to become independent of income assistance and to make significant life changes. Using this "low barrier" approach, virtually any IDU who wants a full-time job and is capable of performing one, is able to secure employment as long as a position is available.

The valued collective knowledge of the peer workers is paramount to the success of the program. They are the eyes and ears, the heart and soul, and are always willing to share their experiences in hopes to improve the program. They are the first to know, for example, if there is a "bad" batch of drugs on the street, if there is a new hotspot for used syringes, and what the specific needs are for themselves as users and for their peers.

## Low threshold and inclusive

The WND is an essential service in promoting harm reduction because it is the only "low threshold" needle distribution program in Vancouver. This means that the program is designed to be completely accessible to all people, both receiving and participating in service. Rooted in public health, in harm reduction the focus shifts from drug use itself to the effects or consequences of addictive behaviour. Harm reduction accepts the fact that many people use drugs and engage in other high-risk behaviours, and that idealistic visions of a drug-free society are unlikely to actually happen. Harm reduction advocates endeavour to reduce the harm associated with drug use, with the possibility of ceasing drug use all together [29].

A low-threshold environment provides opportunities for virtually any individual wishing to become involved. Program Coordinators in the WND report working with many individuals who are not able to participate in service delivery in other programs for a variety of reasons including active addiction, psychiatric or physical health barriers. In addition to creating a diverse service delivery team for the WND; this has the benefit of psychosocial engagement for often marginalized individuals. The WND attempts to create a sense of membership and belonging while promoting safe injection practices.

Many individuals dealing with active drug addictions in the DTES experience daily exclusion based on gender, ethnicity, class, and lifestyle. In this context, VCH strives to provide a continuum of services that meet a wide range of needs in addiction services. To this end the WND is an example of a service that promotes inclusivity as an active component of addiction services. The WND offers paid work for participants regardless of gender, levelling the frequently unequal field of work that regularly finds women and transgendered individuals performing sexualized work in order to pay for their addiction. Because the work is designed to be low threshold, there is no room for exclusion based on ethnicity, class or lifestyle among the paid volunteers. Ethnicity matters, and health care is often 'racialized', meaning that the process of racialization can shape how health providers treat clients or patients [30]. Because the peer workers at the WND come from the DTES and are not discriminated based on ethnicity, they are typically representative of the service population. While there are regular disagreements as in any workplace, generally the WND is able to offer a workplace free from discrimination that respects equally both workers and those receiving service.

## From exchange and centralization to distribution and decentralization

During its first decade of operation from 1988 to 1998, Vancouver's first needle syringe program at DEYAS operated using an exchange model. At that time, the needle exchange program was centralized, that is, ostensibly controlled by one agency. There were set limits on the syringes that were allowed by people recovering from addiction and the process of distribution and retrieval were closely linked in each interaction with IDUs relying on the program. The syringe distribution program of the PHS was the only exception.

**Figure 3:**
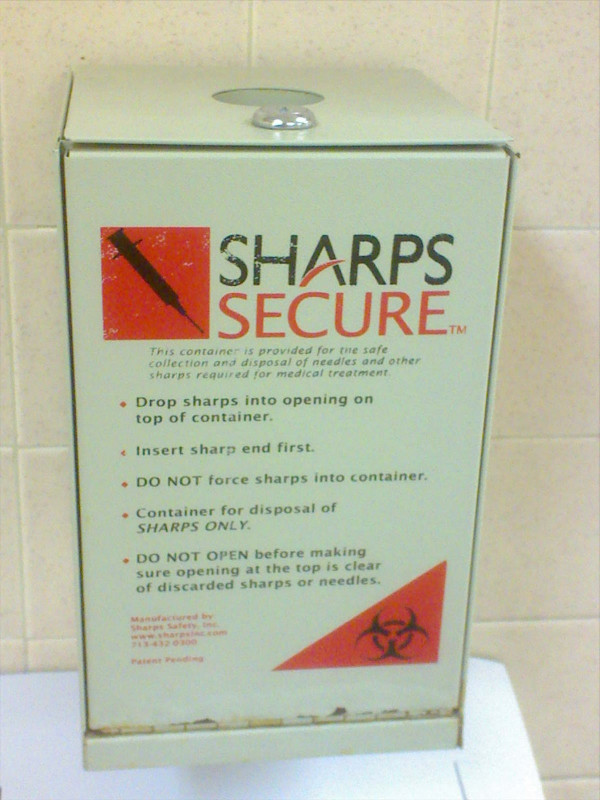
**Syringe receptacle at Seaworld**. A photograph showing a syringe receptacle in the bathroom at the Seaworld public attraction.

In 1999, the health authorities in Vancouver began a process to decentralize needle distribution with a plan to make syringes available through a variety of government clinics and non-profit agencies serving active drug addicts. By the year 2000, the health authority for Vancouver was supervising the distribution of syringes through health clinics, peer support groups, homeless shelters, non-profit agencies and housing providers. This took place against a backdrop of a widespread attempt to place needle disposal boxes in healthcare, housing and public settings. This process of expanding retrieval points for used syringes in public places for needles is not unique to Canada. Today, needle retrieval boxes are located in many public places such as the bathrooms at the famous San Diego SeaWorld attraction (see Figure 3).

**Figure 4:**
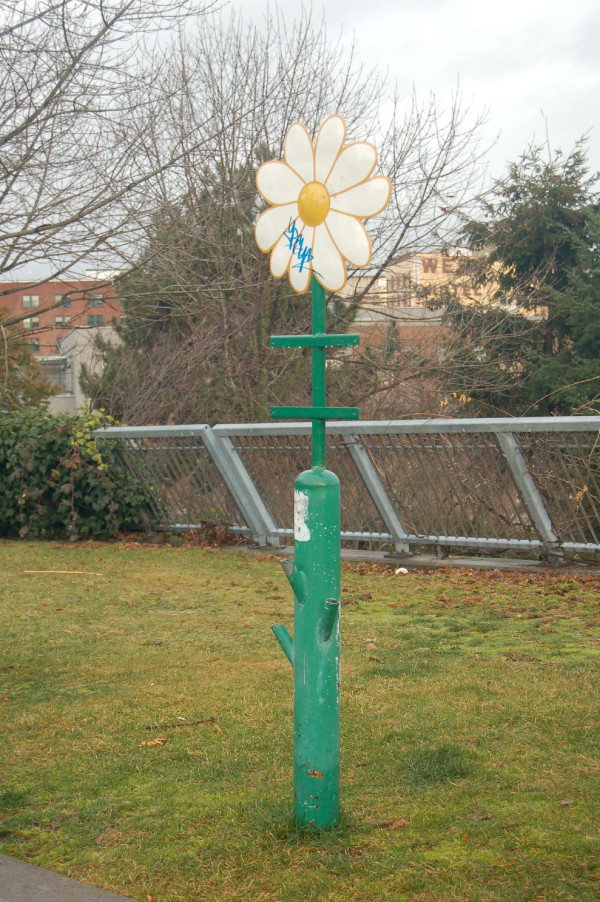
**Daisy receptacle**. A repository for used syringes installed in a Vancouver park.

In fact, a culture change in terms of our understanding about the process of retrieving syringes has occurred in the past ten years in Vancouver. Rather than linking the retrieval process to the point of distribution, the addict, we were separating the process of recovering used syringes from distributing new ones. It has become clear that retrieval of used needles is a practical matter of sanitation and public safety rather than something that has to be tied to needle exchange. This process was taking place at many levels. The City of Vancouver, for example, installed a needle receptacle, in the artful shape of a daisy, in a park adjacent to the Downtown Eastside during this period (see Figure 4). In analogy, if there is a problem with too much garbage in public parks, then it is a suitable public response to install more garbage cans. Similarly, with a goal to recover as many used syringes from the public spaces as possible, there can be increasing resources dedicated to this issue with a practical response: more receptacles for dirty needles and more people paid to pick them up with gloves and tongs. Needle receptacles were placed throughout the public spaces wherever addicts might require them and roving teams called "needle sweeps" were created. The VCH began to keep track of each area of the City of Vancouver as a separate zone to determine "hot spots" where more attention to needle pick-up might be required. Today it is also the standard of practice to install and maintain receptacles to retrieve used syringes within social (government funded) housing in Vancouver.

Underlying the disconnection between distribution and retrieval was a change in our understanding with respect to the *in*effectiveness of straight exchange. The reality is that people who are injecting drugs in unsafe and unclean places are often very wounded people, as indicated by their willingness to purchase illicit substances and inject these substances into their bodies in very unclean and unsafe conditions. This is not to say that personal responsibility cannot be encouraged in the community of drug users, but to highlight the fact that they are at the edge of personal survival, in a kind of "fight or flight" modality. Like most people, their centre of gravity, per se, is not always located around an elaborate planning process for maintaining personal health. If not able to meet the rules of a needle exchange program in order to get sanitary injection equipment, some drug users are more likely to take on additional personal risk (sharing syringes).

The effectiveness of disconnecting distribution and retrieval can be objectively measured. The process is simple: count how many needles were distributed and how many were retrieved? This can be expressed as a percentage sometimes referred to as the "recovery rate". In fact, the recovery rate for the WND is often at 100 per cent (or higher). This is due to the fact that roving teams recover large batches of needles when an IDU drops them off or when a needle retrieval outreach worker pays a visit to the SRA room of an IDU to clear out a large batch (sometimes hundreds) of needles in a single visit. Although the WND sometimes gives out more needles than are returned, there are months where the number of "found" needles combined with the number of "returned" needles surpasses the number of needles that are given out. This highlights the effectiveness of separating retrieval from distribution.

## The Division of Needle Distribution and Retrieval in the 21^st ^Century

Needle exchange and needle distribution are two very different approaches to addressing the spread of HIV and HCV. They are healthcare worlds apart. Needle exchange insists that IDUs exchange dirty needles in order to obtain new needles. There are variations in this approach ranging from strict one-for-one exchange rules to more flexible approaches that allow pre-set amounts of "loaner" syringes. In a one-for-one approach, IDUs simply are not allowed to have a clean syringe unless they have a dirty one to trade. In a more flexible exchange approach, IDUs must, overall, exchange dirty needles for clean ones, but they are allowed, within pre-set limits to borrow clean ones, as "loaners" as long as they return a dirty one at the point of exchange at a later point. These approaches also place limits on the amount of needles that a person can obtain within a given period and, as a result, significantly reduce the impact of NEPs [13].

Various rationales are at the base of exchange approaches to needle programs. The first is that the belief that the retrieval of needles must be embedded within the very practice of distributing needles. Each time an addict receives a needle or a portion of needles, they must simultaneously engage in the process of salvaging the same amount of needles. The process of exchanging syringes is meant to enforce a kind of personal responsibility for people with addictions. This would not be unlike making an alcoholic bring a wine bottle back before they could purchase another bottle of wine. Or, taken out of the addiction realm, it would be like enforcing that each time a person wanted a container of milk, they would have to return an empty milk carton, as opposed to current programs that separate the distribution and recovery of recyclables such as milk cartons and wine bottles.

Secondly, this approach aims to enforce the practice of appropriate disposal of used needles. By providing a kind of "value" to dirty needles, it is expected that people with addictions will keep them in order to obtain new needles. This model is meant to create a kind of positive economy in dirty needles. People with addictions keep the needles in their pockets and rooms so that they can use them as a currency to trade for new needles, despite the obvious health hazards that this entails.

Thirdly, limitation on the number of needles in the exchange model is meant to promote a kind of closeness or rapport between the person that needs the needle and the person that is paid to provide the needle. Compelling the addict to engage the needle provider numerous times every day of every week of their life is meant to provide a link to healthcare services such as detoxification, treatment or counseling. As such, it is a kind of "forced" proximity between healthcare provider as a source of support and referrals and the person in need. In analogy, this approach is similar to a religious organization providing food to the starving but insisting on some participation in religious activities in order to obtain the food. The needle exchange provider becomes a healthcare missionary saving healthcare souls as a condition for receiving the gift: the life saving needle.

In contrast to these three rationales at the base of exchange approaches, needle *distribution *approaches focus primarily on stopping the spread of HIV and HCV transmission by providing as many clean needles as are required. This is achieved by providing IDUs with as many needles as they need so that they have brand new needles and injection equipment for each "fix." This approach is coupled with educating IDUs on HIV and HCV transmission via shared needles so that they are empowered to (a) never share needles (b) return all their used needles to depots or needle disposal boxes, and (c) educate their peers about dangerous injection practices. The distribution approach recognizes that it may not always be possible for IDUs to return every single needle to the location it was dispensed from (e.g. perhaps the mobile van is not nearby). Instead, importance is placed on using needles once only, and on their safe disposal to prevent transmission of disease. This approach does not condone injection drug use; rather the aim is to respond to a public health threat in an effective and respectful manner. A key advantage of this approach is that IDUs are treated with equality that ideally builds trust in the system and allows this vulnerable population to freely access health care services that will save their lives.

It is our experience, through the WND, that the majority of people with addictions will dispose of their syringes appropriately. They do not, by way of example, have to dispose of them through exchange. Many IDUs share the same concerns about community safety as people without addictions. As such, they concern themselves with making sure needles are put into appropriate repositories and that they are not left in public places (such as playgrounds). This is not to say that there are not exceptions, "bad apples" that discard their needles without concern for others. But, these people, consumed by their own needs at the edge of survival, are not the majority. Retrieving needles is a key component of the WND but retrieval is *not *connected, directly, to dispensing syringes.

Sometimes, health authorities embed an exchange ethos into distribution programs. For example, the monthly statistics form for needle distribution from the VCH carries an official "performance target" of 90% written at the top of the form. The separation of syringe retrieval and distribution through the WND results in the retrieval rate (the number of needles collected) relative to the total amount distributed has remained at *over *100% over the past five years of the program operation. That is to say, more syringes are retrieved than distributed, on average, by the WND. This illustrates that a high "retreival percentage" of used syringes can be reached without relying on a strict exchange model.

## The Washington Needle Depot

The WND is innovative in several ways. Firstly, it is a needle *distribution *program rather than a needle *exchange *program- a crucial distinction that goes to the very heart of how needle supply programs are delivered. This paper presents an argument for needle distribution, rather than needle exchange, as a standard of practice. Secondly, the program makes use of a partnership with professionals who work alongside "peers," thus drawing on the experience and street level rapport of people with active addictions while ensuring the service is delivered at optimum levels. Thirdly, the program provides immediate jobs for people who are actively addicted, many of whom are street entrenched. People do not have to go through a lengthy training period or program to participate. They can, in many cases, start the very same day that they arrive from the street. The job is simultaneously part of the recovery process by providing paid employment and validation of peoples' direct life experience in the area where service is being delivered. By providing work immediately, in some days on the very same day that a person shows up to a job meeting directly from the street, the program inverts traditional vocational models that demand that IDUs be living an abstinence based lifestyle before obtaining employment. In essence, rather than getting people ready for work and then eventually giving them employment, the WND gives people a chance work immediately. Work at the WND has a great deal of "symbolic capital" in that its primary purpose is to save lives as opposed to the more menial jobs typically offered to people with long-term barriers in finding employment [31].

The WND draws heavily from the experiential resources of people with active addictions from the community. In its early stages, the program was operated in partnership with a peer support organization for people with addictions (VANDU). Ms. Thia Walter, a feisty activist, advocate and elderly mother whose son struggled with addiction, subsequently volunteered to assist with the recruitment and engagement of street entrenched injection drug users (IDU) as participants in the program.

The involvement of people with active addictions in the provision of harm reduction accomplishes two goals. Firstly, it validates the experience and humanity of an extremely marginalized group of citizens who face multiple obstacles to their social tenure. People with addictions are welcomed into an entry-level role providing life saving healthcare. They have access to a range of "low threshold" vocational opportunities that range from being paid for the day to full time employment. Secondly, the program makes use of the rapport and credibility of people with active addictions to reach extremely marginalized people who live in the shadows of the community. Programs with a peer component can be very effective at reaching marginalized and high-risk IDUs [13,19].

## Politics and Policies

In our view, needle exchange needs to be replaced by needle distribution in every possible instance. Policy makers and professionals are often complicit in all of this, insisting on an exchange to somehow make addicts accountable and forcing points of contact with professionals who sometimes feverishly promote the virtues of treatment and detox [32]. Needle exchange, from this perspective, is a kinder, gentler, approach to enforcement (of community will with respect to how needles are discarded) and treatment (referrals to the healthcare system). Yet, this type of forced exchange would not be tolerated in other healthcare realms outside of addiction. Imagine a situation, for example, where a heart patient or person with cancer had to exchange their chemotherapy pill bottle before receiving a refill. Forced exchange has virtually nothing at all to do with preventing the spread of infectious diseases; it as attempt at imposing the wider community's will upon already marginalized IDUs.

Needle exchange may have been a necessary political stop along the road to adequate harm reduction to address the pandemic of HIV and HCV. In some jurisdictions, even needle exchange is not sanctioned. In the United States, or instance, federal funding for NEPs is not allowed [13]. But now that the evidence base has shown us the effectiveness of syringe distribution programs, we need to eliminate moral and political values that are a barrier to life saving healthcare in this area. We need to acknowledge that enforced and restricted needle exchange is, at its foundation, public policy formed on the basis of the exception rather than the routine, responding to emotive images such as the ever elusive and rare needle that might be hypothetically found (or imagined) in a playground.

Antagonism towards NEPs can lead to increasing restrictive operating policies such as strict exchange policies, daily limits on syringes and reduced hours of operation. Yet, there is a powerful association between high-risk behavior (needle sharing) and problems with adequate access to syringes for IDUs [13]. For instance, enforcement initiatives can have a significant effect on the core operations of NEPs [13,28]. Specifically, police presence can dramatically impact the number of syringes that are distributed through a NEP. Even in places where syringes are legally accessible in Canada, such as pharmacies, the requests of IDUs for these life saving items are often turned down [33]. As obstacles to syringes for IDUs elevate the risk for the spread of infectious diseases like HIV[13], these barriers need to be removed wherever possible. One of the areas where service providers can be a part of the solution is to remove barriers associated with more rigid exchange policies (syringe for a syringe).

## Conclusions

This paper provides an overview of the WND, a program operated by the PHS Community Services Society in Vancouver, British Columbia that shows that the distribution and retrieval of syringes can be separated with effective results. Needle exchanges tend to focus on exchanging clean syringes for dirty ones. However, it is not essential, or necessarily effective, to link clean syringe distribution and the syringe retrieval at the point of contact with IDUs. The WND makes use of the experience of active addicts, through paid employment, to provide an extremely vulnerable population of people with clean syringes to prevent HIV and HCV.

Throughout their history, needles have been employed in healthcare in order to alleviate suffering (e.g. to assist with relieving pain or prevent deadly diseases like HIV or HCV). The WND makes healthcare contact with an extremely hard to reach population of IDUs. To date, there have been over 2 million syringes distributed through the WND and close to 100,000 points of healthcare contact over the six-year span of the program. In order to achieve this, a number of innovations have been built into the program. It is a professional and peer partnership that brings together professional quality assurance and peer-to-peer expertise to reach a difficult target group (those IDUs unconnected to healthcare in any other way). Further, it operates during the most difficult times (between midnight and 10 am) and in the most difficult of places to reach with traditional healthcare (e.g. alleyways and SRA hotels). Unlike more institutional models where IDUs are expected to come to healthcare centres and wait patiently for service, the WNP brings healthcare to IDUs. The program is a decentralized needle exchange (providing needles from a specific location as well as through roving patrols of harm reduction workers), and, equally important, separates the functions of needle distribution and retrieval while removing syringe limits.

The WND is available when, where and how addicts need the program, and is flexible in meeting new needs that arise out of the context of addicts' real, and not imagined lives, where illness and the risk of it exist in the life world of the IDU rather than the clinic. The program operates 24 hours a day with a critical coverage during the late night hours in difficult to reach parts of the inner city. It reaches the most vulnerable addicts, immediately, with healthcare (e.g. clean syringes, referrals to detox and treatment, peer support and first aid) as well as entry-level work opportunities. The WND recruits active addicts directly from the street to be a part of delivering harm reduction services that draw on their skills and experience. The program builds on the experience and rapport of people with active addictions in order to reach a vulnerable population while maintaining high levels of quality assurance with professional oversight. The centre of gravity for syringe distribution programs needs to shift from politics to epidemiology. In order for this to be accomplished, needle exchange needs to be replaced by a needle distribution model (unlimited access to syringes). The WND provides a case study for a needle distribution program with the fundamental goal of fitting itself to injection drug users rather than forcing injection drug users to fit to a program.

## Competing interests

The authors declare that they have no competing interests.

## Authors' contributions

DS wrote the first draft and AG collaborated on subsequent drafts. GR and TW drew on their considerable experience with syringe distribution and retrieval programs to provide observations that strengthened the final version of the paper. All authors read and approved the final manuscript.

## Supplementary Material

Additional file 1 **Open letter written to the Office of National Drug Control Policy**. Letter to Office of National Drug Control Policy from Ranking Member Henry A. Waxman on behalf of the Congress of the United States House of Representatives Committee on Government Reform on 25 May 2005. Click here for file
