# Supplementary material for: The Washington Needle Depot: fitting healthcare to injection drug users rather than injection drug users to healthcare: moving from a syringe exchange to syringe distribution model
Source: Harm Reduct J. 2010 Jan 4;7:1. doi: 10.1186/1477-7517-7-1 (PMC2806876; doi:10.1186/1477-7517-7-1)
Supplement: Additional file 1 — Open letter written to the Office of National Drug Control Policy. Letter to Office of National Drug Control Policy from Ranking Member Henry A. Waxman on behalf of the Congress of the United States House of Representatives Committee on Government Reform on 25 May 2005. [file 1477-7517-7-1-S1.pdf]

TOM DAVIS, VIRGINIA,
CHAIRMAN

CHRISTOPHER SHAYS, CONNECTICUT
DAN BURTON, INDIANA
ILEANA ROS-LEHTINEN, FLORIDA
JOHN M. McHUGH, NEW YORK
JOHN L. MICA, FLORIDA
GIL GUTKNECHT, MINNESOTA
MARK E. SOUDER, INDIANA
STEVEN C. LATOURETTE, OHIO
TODD RUSSELL PLATTS, PENNSYLVANIA
CHRIS CANNON, UTAH
JOHN J. DUNCAN, JR., TENNESSEE
CANDICE MILLER, MICHIGAN
MICHAEL R. TURNER, OHIO
DARRELL ISSA, CALIFORNIA
VIRGINIA BROWN-WAITE, FLORIDA
JON C. PORTER, NEVADA
KENNY MARCHANT, TEXAS
LYNN A. WESTMORELAND, GEORGIA
PATRICK T. McHENRY, NORTH CAROLINA
CHARLES W. DENT, PENNSYLVANIA
VIRGINIA FOXX, NORTH CAROLINA

ONE HUNDRED NINTH CONGRESS

Congress of the United States

House of Representatives

COMMITTEE ON GOVERNMENT REFORM

2157 RAYBURN HOUSE OFFICE BUILDING

WASHINGTON, DC 20515–6143

MAJORITY (202) 225–5074
FACSIMILE (202) 225–3974
MINORITY (202) 225–5051
TTY (202) 225–6852

http://reform.house.gov

HENRY A. WAXMAN, CALIFORNIA,
RANKING MINORITY MEMBER

TOM LANTOS, CALIFORNIA
MAJOR R. OWENS, NEW YORK
EDOLPHUS TOWNS, NEW YORK
PAUL E. KANJORSKI, PENNSYLVANIA
CAROLYN B. MALONEY, NEW YORK
ELIJAH E. CUMMINGS, MARYLAND
DENNIS J. KUCINICH, OHIO
DANNY K. DAVIS, ILLINOIS
WM. LACY CLAY, MISSOURI
DIANE E. WATSON, CALIFORNIA
STEPHEN F. LYNCH, MASSACHUSETTS
CHRIS VAN HOLLEN, MARYLAND
LINDA T. SANCHEZ, CALIFORNIA
C.A. DUTCH RUPPERSBERGER,
MARYLAND
BRIAN HIGGINS, NEW YORK
ELEANOR HOLMES NORTON,
DISTRICT OF COLUMBIA

BERNARD SANDERS, VERMONT,
INDEPENDENT

May 25, 2005

The Honorable John P. Walters
Director
Office of National Drug Control Policy
Executive Office of the President
Washington, DC 20503

Dear Mr. Walters:

Last month, my staff met with staff from the Office of National Drug Control Policy (ONDCP) to discuss needle exchange programs. At this meeting, the ONDCP staff appeared unaware of the extensive scientific evidence and expert opinion that supports needle exchange programs as an effective public health intervention. In fact, your staff asked my staff for references to expert statements that support the effectiveness of these programs.

This letter responds to ONDCP's request for information about needle exchange programs. Since 1991, there have been at least 17 major reviews and assessments of needle exchange programs by expert bodies such as the National Commission on AIDS, the Institute of Medicine, the National Institutes of Health, the Centers for Disease Control, the American Medical Association, the American Society of Addiction Medicine, and the World Health Organization. These assessments have found that needle exchange programs help reduce the spread of AIDS and other dangerous infectious disease without encouraging or increasing drug use. In fact, according to experts, needle exchange programs provide valuable opportunities to reduce illegal drug use.

In part as a result of these conclusions, needle exchange programs have been endorsed by a wide range of expert scientific and medical organizations, including the American Academy of Family Physicians,[1] the American Academy of Pediatrics,[2] the American Academy of Physician

---

[1] American Academy of Family Physicians, *Substance and Alcohol Abuse and Addiction* (2003) (online at http://www.aafp.org/x7096.xml).

[2] American Academy of Pediatrics, Provisional Committee on Pediatric AIDS, *Reducing the Risk of Human Immunodeficiency Virus Infection Associated with Illicit Drug Use,* Pediatrics, 945–7 (Dec. 1994) (online at http://aappolicy.aappublications.org/cgi/reprint/pediatrics;94/6/945.pdf).

Assistants,[3] the American College of Preventive Medicine,[4] the American Medical Association,[5] the American Nurses Association,[6] the American Psychological Association,[7] the Association of Nurses in AIDS Care,[8] and the Infectious Diseases Society of America.[9]

In chronological order, expert reviews and assessments of needle exchange programs include the following:

- In 1991, the **National Commission on AIDS**, whose members included then–Secretary of Defense Richard B. Cheney, convened a hearing on drug use and HIV. Based on the testimonies of expert witnesses at the hearing and a review of scientific evidence, the Commission issued a report that found:

  > Outreach programs which operate needle exchanges and distribute bleach not only help to control the spread of HIV, but also refer many individuals to treatment

---

[3] American Academy of Physician Assistants, *Needle/Syringe Access for the Prevention of HIV Transmission* (policy paper adopted 2003) (online at http://www.aapa.org/policy/needle_access.html).

[4] American College of Preventive Medicine, *Public Policy on Needle-Exchange Programs to Reduce Drug-Associated Morbidity and Mortality*, American Journal of Preventive Medicine, 173–5 (2000) (online at http://www.acpm.org/ajpm369.pdf).

[5] American Medical Association, *Syringe and Needle Exchange Programs* (Policy Statement H-95.958) (online at http://www.ama-assn.org/apps/pf_new/pf_online?f_n=resultLink&doc=policyfiles/HnE/H-95.958.HTM&s_t=syringe+and+needle+exchange+programs&catg=AMA/HnE&catg=AMA/BnGnC&catg=AMA/DIR&&nth=1&&st_p=0&nth=1&).

[6] American Nurses Association, *Position Statement: Needle Exchange and HIV* (effective Apr. 2, 1993) (online at http://nursingworld.org/readroom/position/blood/blnedl.htm).

[7] American Psychological Association, *APA Council Endorses Fair-Testing Code, Forms New Working Group, among Other Actions* (Apr. 4, 2001) (online at http://www.apa.org/monitor/apr04/endorses.html).

[8] Association of Nurses in AIDS Care, *Position Statement: Needle and Syringe Exchange* (rev. Sept. 2002) (online at http://www.anacnet.org/media/pdfs/PS_Needle_Syringe_Exch_4-2003.pdf).

[9] Infectious Diseases Society of America, *Supporting Document for IDSA's Policy Statement on Syringe Exchange, Prescribing and Paraphernalia Laws* (Oct. 5, 2001) (online at http://www.idsociety.org/Content/ContentGroups/Public_Statements_and_Policies1/Statements/Supporting_Document_for_IDSA's_Policy_Statement_on_Syringe_Exchange,_Prescribing_and_Paraphernalia_L.htm).

> programs . … Most significantly, these programs, rather than encouraging substance use, lead a substantial number of substance users to seek treatment.[10]

- In 1995, a report prepared by a joint panel of the **National Research Council** and the **Institute of Medicine** reviewed the available evidence on needle exchange programs and concluded: "Needle exchange programs reduce the spread of HIV — the virus that causes AIDS — without increasing either the injection of illegal drugs among program participants or the number of new initiates to injection drug use."[11]

- In 1997, a Consensus Panel convened by the **National Institutes of Health** concluded:

> An impressive body of evidence suggests powerful effects from needle exchange programs. The number of studies showing beneficial effects on behaviors such as needle sharing greatly outnumber those showing no effects. There is no longer doubt that these programs work . … Does needle exchange promote drug use? A preponderance of evidence shows either no change or decreased drug use.[12]

- In 1997, the Council on Scientific Affairs of the **American Medical Association** issued a report on the medical and scientific literature on needle exchange programs that found:

> There is substantial evidence of reduced needle-sharing among regular participants in needle-exchange programs. More importantly, HIV infection rates among drug users have been consistently lower in cities with needle-exchange programs … . For example, while the HIV infection rate among injection drug users remained 1% to 2% in the Scottish city of Glasgow, where a needle-exchange program was quickly established, it reached 70% in nearby Edinburgh, where the response of government officials was to implement even more stringent controls over injection equipment.[13]

---

[10] National Commission on Acquired Immune Deficiency Syndrome, *The Twin Epidemics of Substance Use and HIV* (July 1991) (online at http://www.dogwoodcenter.org/references/ studies91F.html#RECOMMENDATION%202).

[11] National Academies, *Needle Exchange Programs Reduce HIV Transmission among People Who Inject Illegal Drugs* (Sept. 18, 1995) (online at http://www4.nationalacademies.org/ news.nsf/isbn/0309052963?OpenDocument).

[12] National Institutes of Health, *Interventions to Prevent HIV Risk Behaviors* (Feb. 11–13, 1997) (Consensus Statement No. 104) (online at http://consensus.nih.gov/cons/104/ 104_statement.htm).

[13] American Medical Association, *Report 8 of the Council on Scientific Affairs (A-97)* (June 1997) (online at http://www.ama-assn.org/ama/pub/category/13636.html).

- In 1997, the **American Public Health Association** stated that "an enormous body of published research" exists "attesting to the efficacy of clean needle exchange for reducing HIV transmission among drug users. Moreover, study after study has shown that needle exchange does not lead to an increase of illegal drug use."[14]

- In 2000, the **U.S. Surgeon General** and other senior scientists at the **Department of Health and Human Services** reviewed scientific research completed since April 1998 on needle exchange programs. In a published summary of the review, the Surgeon General announced:

> After reviewing all of the research to date, the senior scientists of the Department and I have unanimously agreed that there is conclusive scientific evidence that syringe exchange programs, as part of a comprehensive HIV prevention strategy, are an effective public health intervention that reduces the transmission of HIV and does not encourage the use of illegal drugs.[15]

- In 2000, the **Institute of Medicine** released a report on the findings of a committee it had convened at the request of the Centers for Disease Control to conduct a comprehensive review of current HIV prevention efforts in the United States. The report described the evidence on needle exchange programs as "compelling" and cited a study that suggested that "expanded provision of needle exchange programs in the United States could have averted between 10,000 and 20,000 new infections over the past decade."[16] According to the report:

> Although many communities and law enforcement officials have expressed concern that increasing availability of injection equipment will lead to increased drug use, criminal activity, and discarded contaminated syringes, studies have found no scientifically reliable evidence of these negative effects.[17]

---

[14] American Public Health Association, *American Public Health Association Supports Lifting Federal Ban on Funding for Needle Exchange* (Aug. 20, 1997) (online at http://www.apha.org/news/press/1997/needle.htm).

[15] U.S. Department of Health and Human Services, *Evidence-Based Findings on the Efficacy of Syringe Exchange Programs: An Analysis of the Scientific Research Completed since April 1998* (Mar. 17, 2000).

[16] Institute of Medicine, *No Time to Lose: Getting More from HIV Prevention*, 114–5 (2000) (online at http://books.nap.edu/books/0309071372/html/1.html#pagetop).

[17] Institute of Medicine, *No Time to Lose: Getting More from HIV Prevention*, 114–5 (2000) (online at http://books.nap.edu/books/0309071372/html/1.html#pagetop).

- In 2000, the **Academy for Educational Development**, in a policy report prepared in collaboration with the **Centers for Disease Control**, observed that much research had been conducted on needle exchange programs. Citing this research, the report concludes that "SEPs [syringe exchange programs] have significant positive effects on preventing adverse health consequences associated with injection drug use and … do not increase drug use or promote the initiation of injection drug use."[18]

- In 2000, the **American Society of Addition Medicine** reported that "[n]eedle exchange programs have been shown to be a crucial component of a spectrum of HIV prevention services to injection drug users, resulting in an effective reduction in the transmission of the Human Immunodeficiency Virus." The Society of Addiction Medicine also reported that "[t]here has not been an increase in drug use or an increase in injection as a route of drug administration as a result of implementation of needle exchange programs, nor has there been demonstration of an increase in contaminated injection equipment in the community."[19]

- In 2002, the **Centers for Disease Control** summarized scientific studies of needle exchange programs. CDC explained: "SEPs have been shown to be an effective way to link some hard-to-reach IDUs [intravenous drug users] with important public health services, including TB and STD treatment. Through their referrals to substance abuse treatment, SEPs can help IDUs stop using drugs. Studies also show that SEPs do not encourage drug use among SEP participants or the recruitment of first-time drug users."[20]

- In 2002, the **National Institute on Drug Abuse** published a research-based guide to preventing HIV in drug-using populations. Concerning needle exchange programs, the guide stated: "Evaluations of these programs indicate that they are an effective part of a comprehensive strategy to reduce the injection drug use-related spread of HIV and other blood-borne infections. In addition they do not encourage the use of illicit drugs."[21]

---

[18] Academy for Educational Development, *A Comprehensive Approach: Preventing Blood-Borne Infections among Injection Drug Users*, A7 (Dec. 2000) (online at http://www.thebody.com/cdc/pdfs/comprehensive-approach.pdf).

[19] American Society of Addiction Medicine, *Public Policy of ASAM: Access to Sterile Syringes and Needles* (adopted Dec. 20, 2000) (online at http://www.asam.org/ppol/ Needle%20Exchange.htm).

[20] Centers for Disease Control, *Syringe Exchange Programs* (Jan. 2002) (online at http://www.cdc.gov/idu/facts/aed_idu_syr.htm).

[21] National Institutes of Health, National Institute on Drug Abuse, *Principles of HIV Prevention in Drug-Using Populations: A Research-Based Guide* (Mar. 2002).

- In 2002, a Consensus Panel convened by the **National Institutes of Health** on management of hepatitis C found that "needle and syringe exchange programs . . . have been shown to be effective in preventing HIV transmission and are likely to be useful for decreasing HCV transmission."[22] The panel recommended: "Institute measures to reduce transmission of HCV among IDUs, including providing access to sterile syringes through needle exchange, physician prescription, and pharmacy sales."[23]

- In 2003, the head of the HIV/AIDS unit of the **International Federation of Red Cross and Red Crescent Societies**, stated: "There is clear scientific evidence that needle exchange programmes work. They help contain the HIV/AIDS pandemic, and in a very cost effective way. Evidence is also clear that these programmes do not promote drug use."[24]

- In 2004, the **Association of State and Territorial Health Officials**, together with the **National Alliance of State and Territorial AIDS Directors**, the **National Association of County and City Health Officials**, and the **Council of State and Territorial Epidemiologists**, stated that "[s]cientific evidence demonstrates that needle exchange programs and pharmacy sales of sterile syringes can be effective public health strategies to reduce the transmission of injection-related HIV infection without increasing drug use."[25]

- In 2004, Dr. Elias A. Zerhouni, Director of the **National Institutes of Health**, wrote a letter to members of Congress in response to an inquiry about the scientific evidence on syringe exchange programs. The letter stated: "A number of studies conducted in the U.S. have shown that SEPs do not increase drug use among participants or surrounding community members and are associated with reductions in the incidence of HIV, hepatitis B, and hepatitis C in the drug-using population."[26]

---

[22] National Institutes of Health, *Management of Hepatitis C: 2002* (June 10–12, 2002) (online at http://consensus.nih.gov/cons/116/091202116cdc_statement.htm#5).

[23] *Id.*

[24] International Federation of Red Cross and Red Crescent Societies, *Spreading the Light of Science: Guidelines on Harm Reduction Related to Injecting Drug Use*, 53 (2003) (online at http://www.ifrc.org/cgi/pdf_pubs.pl?health/hivaids/harm_reduction.pdf).

[25] Association of State and Territorial Health Officials, *Position Statement: Guiding Principles for HIV Prevention* (approved June 18, 2004) (online at http://www.astho.org/policy_statements/HIV%20Position%20Statement.pdf).

[26] Letter from NIH Director Dr. Elias A. Zerhouni to Reps. Henry A. Waxman and Elijah E. Cummings (Oct. 7, 2004).

- In 2004, a policy brief from the **World Heath Organization** discussed the evidence obtained from a review of more than 200 studies on needle and syringe exchange programs. The policy brief reported:

> There is compelling evidence that increasing the availability and utilization of sterile injecting equipment for both out-of-treatment and in-treatment injecting drug users contributes substantially to reductions in the rate of HIV transmission. ... There is no convincing evidence of major unintended negative consequences of programmes providing sterile injecting equipment to injecting drug users, such as initiation of injecting among people who have not injected previously, or an increase in the duration or frequency of illicit drug use or drug injection.[27]

- In 2004, the **Joint United Nations Programme on HIV/AIDS** noted that "[a] review comparing HIV prevalence in cities across the globe with and without needle and syringe programmes found that cities which introduced such programmes showed a mean annual 19% decrease in HIV prevalence. This compares with an 8% increase in cities that failed to implement prevention measures."[28]

As I understand it, ONDCP does not currently support needle exchange programs. I would hope that the information in this letter will change your position.

If your position does not change, I would respectfully request an explanation of the scientific basis of ONDCP's position. In addition, if you believe that any of the sources I have cited are in error or are not reliable, I would request an explanation of the errors that you have identified.

Sincerely,

Henry A. Waxman
Ranking Minority Member

---

[27] World Health Organization, *Policy Brief: Provision of Sterile Injecting Equipment to Reduce HIV Transmission* (2004) (online at http://www.wpro.who.int/NR/rdonlyres/BA463DB4-2390-4964-9D86-11CBABCC9DA9/0/provisionofsterileen.pdf).

[28] United Nations Programme on HIV/AIDS, *2004 Report on the Global AIDS Epidemic*, 85 (June 2004) (online at http://www.unaids.org/bangkok2004/GAR2004_pdf/Chapter4_prevention_en.pdf).
